# Foot Traffic Driven Anthropogenic Activity Alters Phyllosphere Microbial Community Characteristics and Putative Pathogens in Subtropical Urban Green Spaces

**DOI:** 10.3390/microorganisms13112464

**Published:** 2025-10-28

**Authors:** Abdul Baess Keyhani, Wei He, Mingjun Teng, Zhaogui Yan, Monira Fayaz, Zhaohui Peng, Yangyang Zhang, Safir Ahmad Tamim, Xiuyuan Wang, Zemin Han, Pujie Wei, Lei Pan, Pengcheng Wang

**Affiliations:** 1 College of Horticulture and Forestry Sciences, Huazhong Agricultural University, Wuhan 430070, China; weihe@mail.hzau.edu.cn (W.H.); tengmingjun@hotmail.com (M.T.); gyan@mail.hzau.edu.cn (Z.Y.); pengzhaohui2022@126.com (Z.P.); yangyangzhang1993@outlook.com (Y.Z.); safirahmad2661@gmail.com (S.A.T.); w490170459@163.com (X.W.); swxfhzm@163.com (Z.H.); wpj19930916@163.com (P.W.); 2 College of Plant Sciences & Technology, Huazhong Agricultural University, Wuhan 430070, China; monirafayaz92@gmail.com; 3 Hubei Academy of Forestry, Wuhan 430075, China; panlei2008@126.com

**Keywords:** ecosystem functioning, sustainable management, forest, greenbelt, parkland, wetland

## Abstract

Green spaces in subtropical cities are important for providing ecological services that support human well-being and serve as reservoirs for diverse microbial communities, which in turn support ecosystem functions. However, studies on the characteristics of the phyllosphere microbial community and public health risks associated with putative pathogens in various urban green spaces exposed to anthropogenic stress remain limited. To address this gap, we collected leaf samples from forests, greenbelts, parklands, and wetlands across Wuhan, China, and analyzed the bacterial and fungal communities via next-generation sequencing (NGS) techniques. For bacterial and fungal communities, alpha diversity was significantly greater in low-traffic zones than in high-traffic zones. Beta diversity analysis revealed distinct clustering of bacterial and fungal communities according to the urban green space type. Anthropogenic factors (foot traffic) influence green space type to shape microbial community structure, function, and stability, with shifts significantly associated with soil physicochemical properties via Mantel tests and redundancy analysis. The relative abundance of *Enterobacter* and *Enterococcus* was significantly greater in high-intensity parklands (HIPS) and high-intensity greenbelts (HIGS) (41.84, 38.32%), respectively. Our findings provide important information for the sustainable management of urban green spaces by regulating microbial communities, offering new insights into ecosystem health and human well-being.

## 1. Introduction

Urban spaces are rapidly expanding globally, and human populations are shifting from rural to urban areas. This shift increases the demand for material production and human consumption, which can significantly influence land use and ecosystem functions [1,2,3]. Urban green areas have become highly important for human well-being, particularly in China, where the rate of depression reached 36% and the lifetime prevalence of mental disorders among adults was 16.6% in 2019 [4,5]. Urban green spaces offer a crucial avenue for mitigating mental health issues, as engagement with nature reduces stress, anxiety, and depression [6,7,8].

Plant leaves are the primary organs for transpiration and photosynthesis in green plants, and they are also settled with microorganisms that can serve as natural openings connecting soil, water, and atmosphere. The phyllosphere microbiota has substantial ecological service function in biochemical cycles and plant health [9,10]. Therefore, it is ecologically important to understand the phyllosphere microbial community composition, diversity, and functions in various urban green spaces [11,12,13,14,15]. The increased contact between humans and nature-derived microbes appears to be associated with reduced susceptibility to a range of immune-mediated noncommunicable diseases [16]. Urbanization has reduced human interaction with natural microorganisms, and the density of urban green spaces is directly related to the diversity of microorganisms in urban environments [17,18]. Therefore, urban planners should prioritize green space design to maximize the biodiversity and health benefits for residents. By integrating the principles of ecological design, such as diverse plant species and soil management, cities can foster microbial communities within urban green spaces. Ultimately, this approach supports mental health and offers a sustainable urban environment for residents.

Despite the recognized importance of microbial diversity in urban green spaces, there is a significant gap in our understanding of the impact of anthropogenic activity on microbial characteristics and the presence of putative pathogenic bacteria within these spaces. Research on the factors influencing the distribution of phyllosphere microorganisms in various urban green spaces is limited. Urban green spaces, including forests, greenbelts, parklands, and wetlands, differ in their socio-ecological characteristics, such as management practices and vegetation communities [19]. Forests provide a diverse range of essential ecosystem services, including, but not limited to, carbon sequestration, oxygen production, climate regulation, and biodiversity conservation [20,21,22]. Greenbelts function primarily as mitigators of road dust and increase the deleterious impact of vehicle exhaust on air quality [23,24]. Parklands cater to the recreational needs of city dwellers by providing favorable settings for physical activity [25]. Wetlands act as natural buffers that control floods, regulate local climates, and provide a great sense of birdwatching [26,27]. These dissimilarities may lead to spatially associated microbial communities that maintain the balance of ecosystems and homeostasis in the human body. Different urban green spaces require distinct management strategies, and understanding phyllosphere microbial diversity across various types of urban green spaces could lead to precision management, thereby improving soil quality and enhancing the health benefits of urban green spaces.

As a proxy for anthropogenic activity in various urban green spaces, the human population could also impact phyllosphere microbial communities. Urbanization, characterized by an increase in the human population and activity, often has complex effects on microbial diversity and richness [10,28]. Notably, these findings are ambiguous. These findings focus primarily on forests, the most diverse community; however, microorganisms in phyllosphere are often overlooked, and the associations between anthropogenic activity and microbial biodiversity in Wuhan, as an important model subtropical city in central China, remain unknown. Specifically, there is a knowledge gap regarding the associations among phyllosphere microbial community characteristics, pathogenic bacteria, and anthropogenic activity in different urban green spaces.

Engaging in outdoor activities within urban green spaces, such as jogging, picnics, and playing, certainly exposes individuals to plants and soil, which impacts microbial communities and provides a pathway for microbial transfer from urban green spaces to humans. Thus, understanding the effects of anthropogenic activity in urban green spaces on microbial community characteristics and putative pathogens requires a detailed characterization of human activity, land use, and physicochemical properties. Wuhan, an important city in Central China with a rich history of industrial activity and investment in forests, public parks, greenbelts, and wetlands, presents a unique opportunity for study. Elucidating the influence of anthropogenic factors, such as human traffic intensity, on microbial characteristics and putative pathogens can offer valuable insights into the management of urban green spaces and contribute to our understanding of their ecological ecosystem health.

Therefore, we evaluated and characterized phyllosphere microbial communities and putative pathogenic bacteria in different urban green spaces in Wuhan via next-generation sequencing (NGS). The physicochemical properties of the soil were also determined and correlated with the microbial communities in each green space because soil physiochemical properties also play an important role in shaping the microbial community characteristics on phyllosphere they influence nutrient availability, moisture, and overall health of the plants, providing more comprehensive perspective on the plant-microbe interaction within ecosystems [29].

This study aimed to (1) assess the influence of human activity on phyllosphere microbial composition, function, diversity, and structure in various types of urban green spaces in a subtropical city; (2) reveal the composition of putative potential pathogens and the public risk in subtropical urban green spaces; and (3) determine the most important physicochemical property factors shaping microbial characteristics within various urban green spaces.

We hypothesized that (1) high-traffic zones in subtropical urban green spaces have distinct microbial communities compared with low-traffic zones; (2) putative pathogens may be related to environmental factors and anthropogenic activity; and (3) microbial composition, richness, and evenness may be strongly related to soil physicochemical properties in various types of green spaces. Our study provides valuable insights into the management of urban green spaces and contributes to our understanding of their ecology and health status.

## 2. Materials and Methods

### 2.1. Sampling and Experimental Design

**Figure 1:**
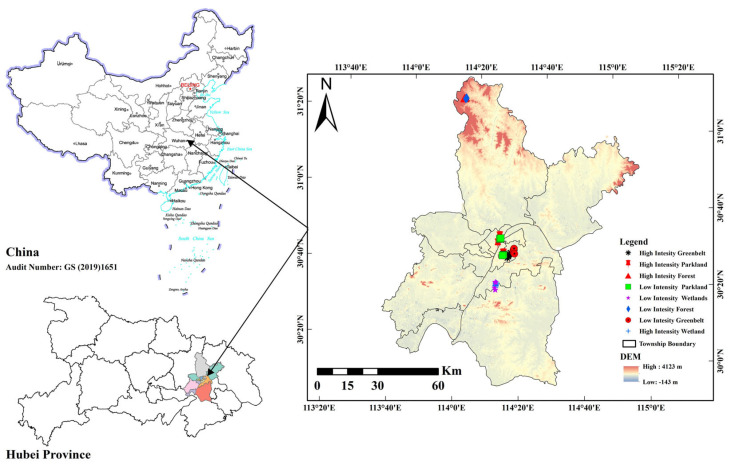
Study area location different shapes represent different urban green spaces.

In this study, foliage samples were collected from 8 sites across Wuhan (30°35′ N, 114°18′ E), China, in May 2024 (Figure 1, Table S1). The weather was sunny, there was no rainfall during sampling, and the temperature ranged from 19 °C to 27 °C during the daytime. The sampling sites were selected on the basis of the level of human traffic at each site, and each sampling site covered 500 m^2^. The sites included forestland (2 sites), greenbelts (2 sites), parkland (2 sites), and wetland (2 sites). Each site was divided into two zones (high and low intensity) on the basis of the level of human foot traffic intensity. The human flow at each site was quantified via anonymized data from the Wuhan Municipal Bureau of Forestry and Landscaping, which were collected via a video surveillance system at green space entrance counters in 15 May 2024 (https://ylj.wuhan.gov.cn/). High-intensity zones were defined as areas with frequent human presence and visible signs of foot traffic (~100–500 human flow ha^−1^ day^−1^), whereas low-intensity zones were more secluded and less populated areas (~0–50 human flow ha^−1^ day^−1^). These sites were chosen to represent the typical vegetation cover and urban green space types in the local area. Each site varies in total extension. Forests and greenbelts are semi-managed areas located near the urban periphery, with minimal human intervention and predominantly native vegetation. Parklands are centrally located, highly managed spaces with a mix of introduced ornamental plants and native species, designed for recreational use. Wetlands are situated near industrial zones and natural water bodies, featuring a combination of restored native vegetation and planned features for flood control and habitat support. Sites selection considered micro localization relative to industrial areas and wild zones to minimize external environmental influences.

Leaf samples of *Sagittaria sagittifolia, Nerium indicum, Nelumbo nucifera, Phragmites australis,* and *Bougainvillea*, which are prevalent in urban green spaces across Wuhan, were collected from aboveground via sterilized scissors and combined into a single sample for further analysis. Species selection was based on their natural occurrence and dominance within each green space type. The topsoil was collected to a depth of 15 cm at the same time. At each site, five leaf and five soil samples were collected. In total, 48 leaves and 48 soil samples (8 sites × 3 replicates × 2 traffic zones) were collected. The initial properties of the samples were characterized in May 2023 (Table S2). All samples were collected after the vegetation roots were removed using sterilized tweezers and then standardized in the laboratory. Subsamples (0.5 g) were placed in sterilized tubes and stored at −80 °C for DNA extraction. All the soil samples were crushed and sieved (2 mm) to remove debris and stone material prior to analysis of the physicochemical properties of the soil.

### 2.2. Determination of Soil Physicochemical Properties

The soil pH was measured in water via a pH meter (Mettler-Toledo, Greifensee, Switzerland). The soil bulk density (g/cm^3^), moisture (%), capillary porosity (%), and noncapillary porosity (%) were measured according to the methods of De Vos et al. and Spasić et al. [30,31]. Available nitrogen (AN) was measured via alkaline hydrolysis diffusion [31]. The total nitrogen (TN) content was measured via the Kjeldahl method (KDN–102C, Shanghai, China), and the total phosphorus (TP) was measured using an elemental analyzer AUTCHEM 1200 elemental analyzer (Changchun Xingrui, China). Available phosphorus was measured via flame photometry. Available potassium (AK) was measured via ammonium acetate, and total potassium (TK) was analyzed via flame photometry after melting with sodium hydroxide, as described by [32]. The soil organic carbon content was measured after digestion with potassium (K_2_Cr_2_O) and FeSO_4_ titration [33].

### 2.3. DNA Extraction, PCR Amplification, Sequencing, and Data Processing

In accordance with manufacturer’s protocol, microbial DNA was extracted from 0.5 g of 56 leaf sample via a DNA extraction kit (M5635-02; Omega Bio-Tek, Norcross, GA, USA). The absorbance of the extracted DNA was measured via a NanoDrop NC200 spectrophotometer (Thermo Fisher Scientific, Waltham, MA, USA). The DNA from these samples was stored at −80 °C until use. The composition of the bacterial and fungal communities was characterized by amplification of the hypervariable V3–V4 region of the 16S bacterial rRNA gene (338F: ACTCCTACGGGAGGCAGCA and 806R: GGACTACHVGGGTWTCTAAT) [34] and the ITS region for fungi (ITS1F: CTTGGTCATTTAGAGGAAGTAA and ITS2: GCTGCGTTCTTCATCGATGC) [35]. The PCRs (25 μL total volume) contained 5 μL of 5× buffer, 0.25 μL of FastPfu DNA polymerase (5 U/μL), 2 μL dNTPs (2.5 mM), 1 μL each of forward and reverse primers (10 μM), 1 μL of DNA template, and 14.75 μL of ddH_2_O. The thermal cycling protocol consisted of an initial denaturation step at 98 °C for 5 min, followed by 25 cycles of denaturation at 98 °C for 30 s, annealing at 53 °C for 30 s, and extension at 72 °C for 45 s, with a final extension of 5 min at 72 °C. PCR amplicons were purified via Vazyme VAHTSTM DNA Clean beads (Vazyme, Nanjing, China) and quantified via a Quant-iT PicoGreen dsDNA Assay Kit (Invitrogen, Carlsbad, CA, USA). After individual quantification, the amplicons were combined in equimolar ratios and sequenced on an Illumina NovaSeq platform (2 × 250 bp paired-end; NovaSeq 6000 SP reagent kit (Shangahi, China), 500 cycles) at Shanghai Personal Biotechnology Co., Ltd. (Shanghai, China).

### 2.4. Bioinformatics and Statistical Analysis

The raw sequence data were quality-filtered and demultiplexed via the QIIME2 v2022.11 microbiome bioinformatics platform. Quality filtering, denoising, and merging were performed via the DADA2 plugin [36]. Non-singleton amplicon sequence variants (ASVs) were aligned via MAFFT [37] and used to construct a phylogeny using fastTree2 [38]. The sequence data were deposited in the National Center for Biotechnology Information (NCBI) Sequence Read Archive under the accession numbers PRJNA1338734 and PRJNA1338651. The soil physicochemical properties of the samples were examined via one-way analysis of variance (ANOVA) (IBM SPSS Statistics for Windows, Version 22. IBM Corp, Armonk, NY, USA), Venn diagram in R package (v3.2.1) was used to construct the number of shared OTUs in the different urban green spaces. Bacterial and fungal alpha diversity were calculated via QIMME. The Chao1, observed species, Shannon, and Simpson diversity indices were used to estimate the richness and diversity of the bacterial and fungal communities separately, and the results were visualized as box plots. Beta diversity was analyzed via Bray–Curtis dissimilarity unweighted UniFrac metrics [39]. Principal coordinate analysis (PCoA) was performed via R package (v3.2.1), with statistical significance tested using PERMANOVA with 999 Monte Carlo permutations based on Bray–Curtis dissimilarity metrics. Taxonomic composition and abundance were visualized via MEGAN [40] and GraPhlAn [41]. Linear discriminant analysis effect size (LEfSe) was performed to detect differentially abundant taxa across groups using the default parameters [42]. Co-occurrence networks were constructed via in Origin Pro 2022 (OriginLab Corporation, Northampton, MA, USA). Species-level abundance profiles were obtained to determine the microbial index of pathogen (MIP) for each microbiota via microbial index of pathogen:

$$\mathrm{MIP}\;=\;\frac{\sum_{i=1}^{M}pathogens\;i}{\sum_{j=1}^{N}mj}$$

where *M* is the number of pathogens in a sample; *N* is the number of all microbes identified in a sample, and *m* is the relative abundance of a microbe in a sample. The microbial index of pathogen (MIP) was calculated as the sum of the relative abundance of all pathogenic bacteria in a microbial community according to 300 published categories of pathogenic bacteria by the Chinese Center for Disease Control and Prevention, ranging from 0 to 1 [43,44]. It is important to note this classification is based on a specific pathogen, meaning their pathogenicity is often context dependent and not globally defiance. The correlations between environmental factors and the composition and diversity of the microbial community were evaluated via the Mantel test. The redundancy analysis (RDA) function in Origin Pro 2022 (OriginLab Corporation, Northampton, MA, USA) was used to examine the possible relationships between environmental variables and pathogenic microorganisms via multiple correlations.

## 3. Results

### 3.1. Description of the Microbial Community

In this study, we selected sequences from various sets and paired them with the corresponding ASVs to generate rarefaction curves for bacteria and fungi (Figure S1a,b). The curves tended to become uniform as the number of sequences increased, revealing higher ASV richness plateaus in specific urban green spaces. The sequencing of eight groups yielded 819,727 raw reads for bacteria, of which 763,262 low-quality sequences were subsequently discarded. Following the denoising process, 722,464 effective sequences were identified, with 575,010 remaining after splicing. After demultiplexing, filtering, denoising, read merging, and chimera removal, the 48 samples contained 528,579 high-quality sequence reads suitable for analysis. The classification identified 166,986 ASVs for bacteria (Table S3).

For fungi, sequencing of the same 48 samples generated 876,257 raw reads. After removing 792,667 low-quality sequences and declassifying, 787,827 effective sequences were identified, with 757,790 remaining after sequence alignment. After demultiplexing, filtering, denoising, read merging, and chimera removal, the samples contained 699,035 high-quality sequence reads that were suitable for analysis. The classification process identified 12,362 unique ASVs for fungi (Table S4).

**Figure 2:**
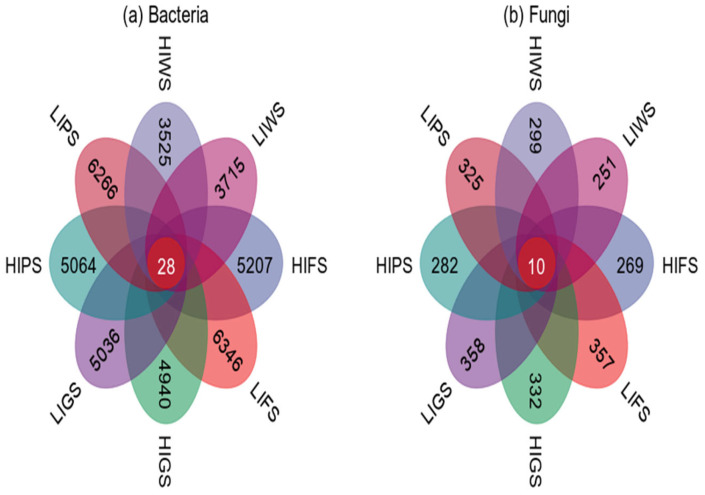
The Venn diagram represents unique and shared bacterial phyla (**a**) and fungi (**b**) across urban green spaces. Green space types include forests (FS), greenbelts (GS), parklands (PS), and wetlands (WS), with high-intensity (HI) and low-intensity (LI).

Venn diagrams were used to visualize the similarities and overlaps between the bacterial and fungal taxa in the various urban green spaces. In this study, we identified 5207, 6346, 4940, 5036, 5064, 6266, 3525, and 3715 unique bacterial species in HIFS, LIFS, HIGS, LIGHS, HIPS, LIPS, HIWS, and LIWS, respectively. Notably, only 28 ASVs were common to all the green spaces. LIFS and LIPS presented the greatest number of unique ASVs (Figure 2a). For fungi, we identified 269, 357, 332, 358, 282, 325, 299, and 251 unique ASVs across the different green spaces, with only 10 common taxa (Figure 2b). The LIGS and LFS presented the greatest number of unique ASVs.

### 3.2. The Composition and Structure of Microbial Communities in Various Urban Green Spaces

**Figure 3:**
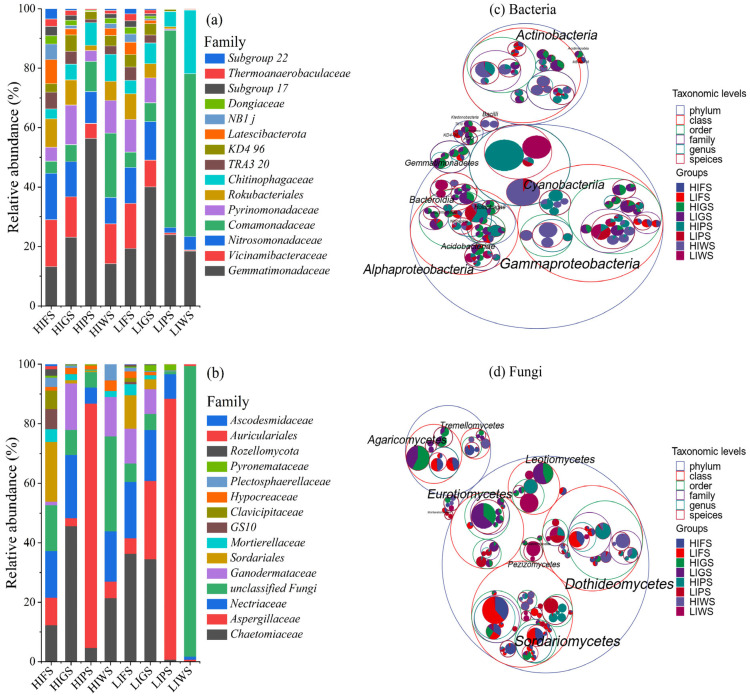
Figures (**a**,**b**) representing the relative abundance of bacteria and fungi in various urban green spaces tree diagram (**c**,**d**) illustrating the microbial communities associated with various urban green spaces. The outermost concentric circle represents the phylum level, with progressively smaller circles indicating the class, order, family, genus, and species, arranged according to the “highest taxonomic level” parameter. Taxonomic levels were differentiated using standard color schemes. The innermost dots represent the top 100 most abundant ASVs/OTUs, with the dot size (area) corresponding to their relative abundance in each group. The size of the circle segments at each taxonomic level also reflects the relative abundance of their respective taxonomic units. Refer to Figure 1 for abbreviations of green space types.

The microbial composition varied significantly among urban green spaces, with bacterial and fungal families and distinct responses, which were dominant at different anthropogenic levels compared to the initial time (Figure 3a,b and Figure S2a,b, respectively). Comamonadacea were dominant at different urban green spaces (LIPS: 92.75%, LIWS: 78.12). Gemmatimonadaceae were also prevalent in low- and high-intensity zones (HIPS: 56.32% and LIGS: 40.07%), whereas the relative abundance of Gemmatimonadaceae markedly reduced at (HIWS: 14.23%, HIFS: 13.2%). Nitrosomonadaceae, pyrinomonadaceae, Rokubacterale, Chitinophasgaceae were most abundant in high-intensity areas but declined in low-intensity areas. Other bacterial families, such as Thermoanaerobaculaceae, Dongiacea, NB1 J, Latescibacterota, remained at low abundance in all urban green spaces. Fungal community analysis revealed *Chaetomiaceae* as the predominant family, particularly at high-intensity zone (HIGS: 45.58%) and low-intensity zone (LIFS: 36.25%), whereas Aspergillaceae was most abundant at the LIPS (88.40%) and HIPS (86.76%). Ascodesmidaceae and other families were observed at low relative abundance throughout the urban green spaces.

To deepen into the taxonomic differences across urban green spaces in high- and low-traffic zones and identify the specific microbial taxa and their taxonomic relationships, a taxonomic tree from phylum to species was constructed using packed circles to identify the top 20 genera (Figure 3c,d). Actinobacteria and Gammaproteobacteria were the dominant bacterial classes, with Actinobacteria being more abundant in low-intensity forests (LIFS) and Gammaproteobacteria being more abundant in high-intensity wetlands (HIWS). Other notable bacterial classes included Alphaproteobacteria, Acidobacteria (particularly low-intensity greenbelts (LIGS), and *Cyanobacteriota*. Fungal communities were primarily composed of Dothideomycetes, which were enriched in LIFS. *Agaricomycetes* were more abundant in high-intensity wetland areas within wetlands (HIWS), and Sordariomycetes, Eurotiomycetes, and Mortierellomycetes presented more localized distributions. In contrast, decomposer taxa, including Actinobacteria and Dothideomycetes, were more prevalent in low-intensity areas.

LEfSe identified 83 bacterial and 12 fungal clades as significant biomarkers (LDA > 3.0). At the class level, Blastochatellia, Holophagae, Thermoanaerobaculia, Vicinamibacteria, Acidobacteriota, and Rubrobacteria were identified as potential bacterial biomarkers, demonstrating significant enrichment in various urban green spaces (Figure S2a). Similarly, Laboulbeniomycetes, Lecanoromycetes, Agaricomycetes, Cystobasidiomycetes, Spizellomycetes, and Mortierellomycota were identified as potential fungal biomarkers significantly enriched in the corresponding green spaces (Figure S3b). High LDA scores indicated group-specific signatures, including Vicinamibacteria (LDA = 4.8), Holophagae (LDA = 4.2), Gemmatimonadetes (LDA = 4.7), Actinobacteria (LDA = 4.2), Verrucomicrobiae (LDA = 3.8), Actinobacteria (LDA = 4.7), Bacilli (LDA = 3.1), and Latescibacteria (LDA = 4.1), observed in HIFS, HIGS, LIGS, HIPS, LIPS, HIWS, and LIWS for bacteria. Among the fungi, Eurotiomycetes (LDA = 5.4), Kickxellomycetes (LDA = 4.9), and Sordariomycetes (LDA = 4.8) predominantly characterized HIWS, HIFS, and LIFS, respectively, reflecting their distinct ecological roles in the forest.

### 3.3. Microbial Diversity

#### 3.3.1. Alpha Diversity

**Figure 4:**
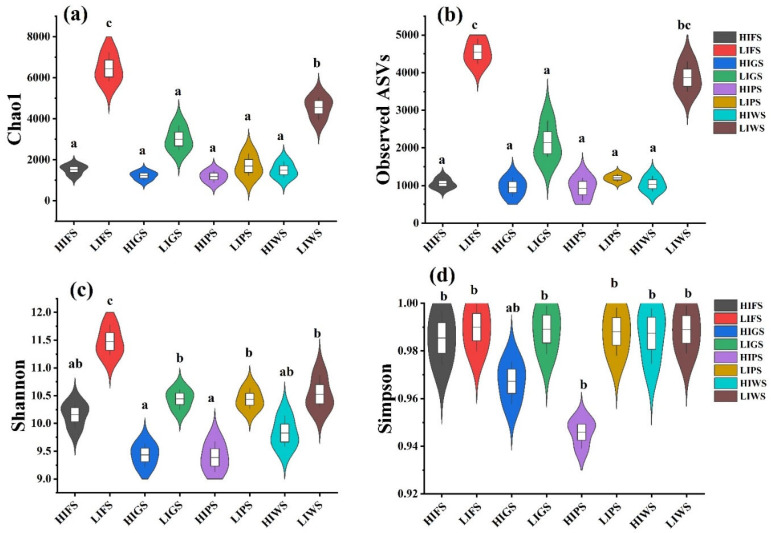
Alpha diversity indices inverse Chao1 (**a**), observed species (**b**), Shannon (**c**), and Simpson (**d**) for bacterial communities in urban green spaces, reflecting species richness and evenness. Different letters indicate statistically significant differences between the groups (*p* < 0.5). Green space types include forests (FS), greenbelts (GS), parklands (PS), and wetlands (WS), with high-intensity (HI) and low-intensity (LI).

**Figure 5:**
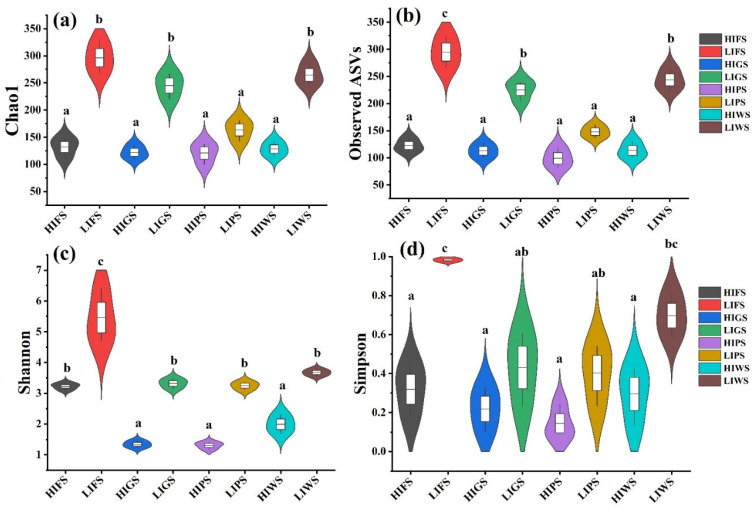
Alpha diversity indices inverse Chao1 (**a**), observed species (**b**), Shannon (**c**), and Simpson (**d**) for fungal communities across urban green spaces, reflecting species richness and evenness. Different letters indicate statistically significant differences between the groups (*p* < 0.05). Refer to Figure 1 for abbreviations of green space types. Green space types include forests (FS), greenbelts (GS), parklands (PS), and wetlands (WS), with high-intensity (HI) and low-intensity (LI).

Alpha diversity indices, including the Chao1, observed species, Shannon, and Simpson indices, were used to assess bacterial and fungal richness and evenness in the urban green spaces. The alpha diversity of the bacterial communities was significantly greater in the low-intensity zones (LIFS and LIWS) than in high-intensity zones (HIGS and HIWS) (*p* < 0.05). Among the different types of urban green spaces, the alpha diversity was notably greater in the LIFS and LIWS (Figure 4). A similar result was observed for fungal communities, where alpha diversity was significantly greater in low-traffic zones than in high-intensity zones (*p* < 0.05). The number of OTUs was considerably greater in the LIFS, LIWS, LIGS, and LIPS groups (Figure 5).

#### 3.3.2. Beta Diversity

**Figure 6:**
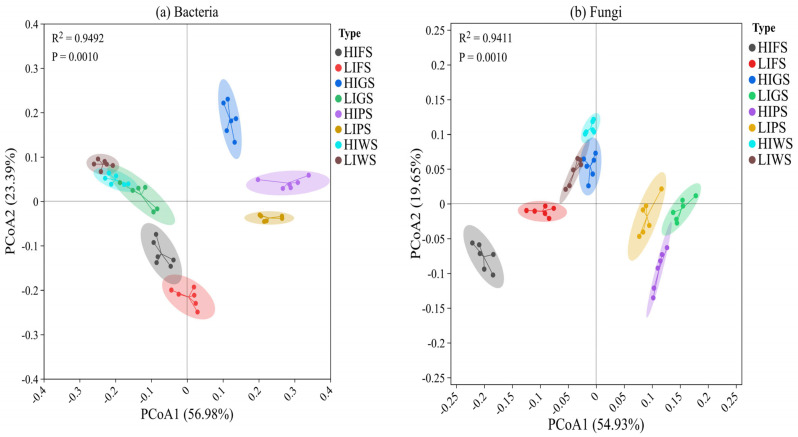
Principal coordinate analysis (PCoA) based on UniFrac distances (unweighted) at different urban green spaces for bacteria (**a**) and fungi (**b**) Principal coordinate analysis (PCoA) based on UniFrac distances (unweighted) at different urban green spaces for bacteria (**a**) and fungi (**b**). Green space types include forests (FS), greenbelts (GS), parklands (PS), and wetlands (WS), with high-intensity (HI) and low-intensity (LI). Green space types include forests (FS), greenbelts (GS), parklands (PS), and wetlands (WS), with high-intensity (HI) and low-intensity (LI).

Principal coordinate analysis (PCoA) was performed to identify heterogeneity among green spaces. Our findings revealed distinct beta diversity patterns for bacterial and fungal communities across urban green spaces (Figure 6a,b). For bacteria, the first two principal coordinates reported a considerable proportion of the total variance (PCoA1: 56.98%, PCoA2: 23.39%), with samples forming well-defined clusters corresponding to high and low human traffic intensities (Figure 6a). Differentiation among urban green spaces was statistically confirmed by PERMANOVA (R^2^ = 0.9492, *p* = 0.001), indicating that 94% of the variance in bacterial community structure could be attributed to the sample type. In contrast, fungal communities displayed lower compositional differentiation across the sample types (Figure 6b). The first two axes of the PCoA explained 54.93% and 19.65% of the variance, respectively, with sample clustering by type being more pronounced than that of the bacterial communities. PERMANOVA results (R^2^ = 9411, *p =* 0.001), indicated that the sample type explained more than 94% of the differences observed in the fungal community composition.

### 3.4. Relative Influence and Contribution of Environmental Factors to Microbial Community Distribution

Mantel tests revealed significant relationships between soil physicochemical properties, microbial diversity, and community structure indices across different urban green spaces (Figure S4). Bacterial diversity (16S_Shannon) and fungal diversity (ITS_Shannon) responded differently to the environmental factors. Bacterial diversity was significantly associated with pH, available nitrogen (AN), available phosphorus (AP), total potassium (TK), carbon to nitrogen (C:N), carbon to phosphorus (C:P), nitrogen to phosphorus (N:P), soil density (SD), and non-capillarity porosity (NCP) at HIGS, LIGS, HIFS, HIWS, and LIWS (0.01 < *p* ≤ 0.5). In contrast, fugal diversity (ITS_Shannon) was significantly correlated with pH, available potassium (AK), total potassium (TK), soil moisture (SM), and carbon-to-phosphorus ratio (C:P) at HIPS and LIPS (0.01 < *p* ≤ 0.5). However, it showed no significant correlation with the environmental factors at HIFS, LIFS, HIGS, LIGS, HIWS and LIWS (*p* > 0.05). Similarly, bacterial community structure (16S_OTUs) exhibited significant relationship with non-capillarity porosity (NCP), and pH at HIFS and LIFS (0.01 < *p* ≤ 0.5). Fungal community structure (ITS_OTUs) was significantly associated with capillarity porosity (C:P), pH, carbon to nitrogen, nitrogen to phosphorus (N:P), and total potassium at HIFS, LIFS, and HIWS (0.01 < *p* ≤ 0.5).

### 3.5. Characterization of Putative Pathogens in Urban Green Spaces and Co-Occurrence Network Dynamics of Microorganisms

**Figure 7:**
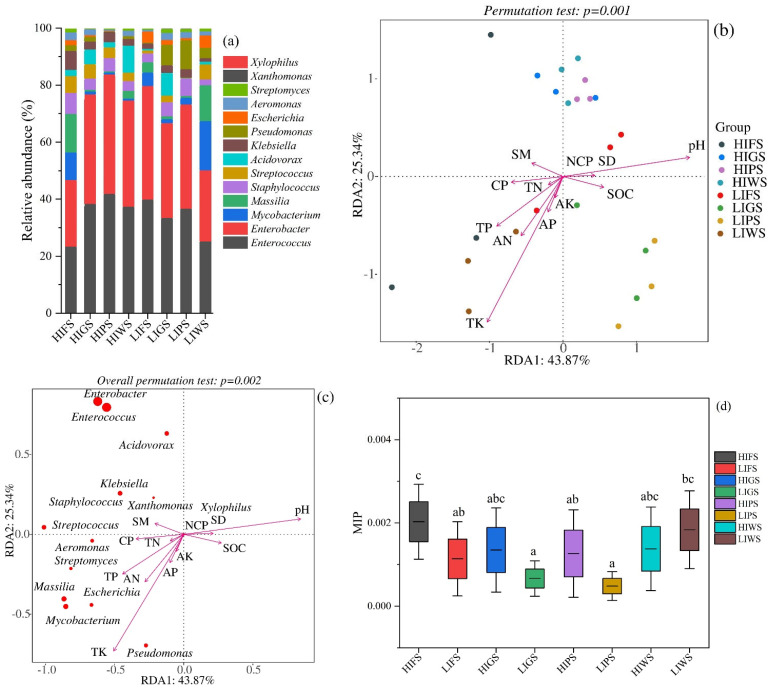
Represents the relative abundance of putative pathogens and the microbial index of pathogenic bacteria in the urban green spaces. (**a**) Relative abundance of putative pathogens in urban green spaces; (**b**,**c**) Redundancy analysis (RDA) of the relative contributions of environmental variables to pathogens. The soil variables include the following: Available nitrogen (AN); Total nitrogen (TN); Available phosphorus (AP); Total phosphorus (TP); Available potassium (AK); Soil organic carbon (SOC); pH; Soil moisture (SM); Soil bulk density (SD); Capillary porosity (CP); and Non-capillary porosity (NCP). (**d**) Comparison of MIP among various types of urban green spaces. Green space types include forests (FS), greenbelts (GS), parklands (PS), and wetlands (WS), with high-intensity (HI) and low-intensity (LI). Different letters indicate statistically significant differences between groups (*p* < 0.05).

To assess potential public health risks in urban green spaces, putative pathogens were identified in foliage via a pathogen detection pipeline and multiple bacterial pathogen detection methods that can detect a broad range of pathogens [45]. The dominant pathogenic genera, including *Enterobacter* (HIPS: 41.84%, LIFS: 39.82%, HIGS: 38.32%) and *Enterococcus* (HIPS: 41.89%, LIFS: 39.88%, LIPS: 36.69%), were significantly more abundant in high-traffic zones than in low-traffic zones, whereas *Staphylococcus* and other pathogens were present at lower abundances (Figure 7a). Redundancy analysis (RDA) revealed that environmental variables, particularly soil moisture and nutrient availability, significantly influenced the distribution of pathogenic bacteria (permutation tests, *p* = 0.001, 0.002) (Figure 7b,c). Soil moisture was a major factor influencing the variation in the abundance of *Staphylococcus* and *Klebsiella* species in this study. Streptococcus was associated with the total soil nitrogen content. The total soil contents of phosphorus, nitrogen, and potassium affected the growth of *Aeromonas*, *Streptomyces*, *Mycobacterium*, *Massilia*, and *Pseudomonas* (Figure 7c). The microbial index of pathogenic bacteria (MIP) has been used to evaluate potential public health risks in various urban green spaces. The MIP values ranged from 0.0026 to 0.0015, with a mean of 0.001 ± 0.00013. There were significant differences in the MIP values among the types of urban green spaces, except for high-intensity greenbelts (HIGS) and high-intensity wetlands (HIWS) (Figure 7d). Our results suggest that physicochemical properties influence the presence of pathogenic bacteria under both high and low anthropogenic activity.

The co-occurrence network analysis of top 15 bacterial and fungal classes revealed distinct patterns between high- and low-intensity urban green spaces (Figures S5 and S6). Bacterial networks exhibited greater overall connectivity and modularity than fungal networks. Within the phyllosphere, bacterial networks complexity was higher in low- intensity zones for all green space types except wetlands. Conversely, fungal network complexity was consistently higher in low-intensity zones across all urban green spaces, including forests, parklands, wetlands, and green belts.

## 4. Discussion

Phyllospheres in urban green spaces facilitate exchanges between natural environmental microbiota. However, previous studies have neglected to assess how anthropogenic activities reshape microbial communities and impact human health despite providing unique opportunities for interactions between humans and the environmental microbiota. In this study, we characterized the composition, structure, and potential pathogens of microbial communities in various types of urban green spaces. Our results demonstrate that anthropogenic activities significantly influence microbial community structure and diversity. Human activities and green space type are the factors putatively driving the physicochemical properties and diversity of microbial communities.

The microbial communities in the phyllosphere of different urban green spaces consistently dominated phyla, including Proteobacteria, Actinobacteria, Ascomycota, Basidiomycota, and Rozellomycota, across urban green spaces [10,46,47,48]. The relative abundance of major phyla, such as Proteobacteria, was significantly greater in the high- and low-human-traffic zones across the wetlands. In contrast, their relative abundance was markedly reduced in low-intensity traffic zones, which is consistent with the findings of previous studies. Proteobacteria are abundant in the phyllosphere [47,49]. Our study demonstrated that human traffic intensity drives pronounced differences in microbial community composition across urban green spaces. High-traffic zones were dominated by stress-tolerant taxa, such as Gammaproteobacteria and Basidiomycota, whereas low-traffic zones favored decomposer groups, such as Actinobacteria and Dothideomycetes. These findings are consistent with global observations of microbial responses to anthropogenic disturbances [28,50]. For example, the dominance of Proteobacteria in high-traffic areas mirrors their metabolic versatility under disturbed conditions, as documented in urban ecosystems [51]. Conversely, the decline in Acidobacteriota abundance in high-traffic areas supports cross-biome findings that disturbance reduces oligotrophic taxa that rely on stable organic matter inputs [28]. The dominance of Ascomycota at high-traffic sites likely reflects their documented resistance to pollutants and compacted soil [52], whereas the abundance of *Actinobacteria* in low-traffic zones in forests corroborates their established role in lignocellulose decomposition in undisturbed ecosystems [53]. Notably, our findings uniquely identified Agaricomycetes as bioindicators of high-traffic zones in wetlands, in contrast to [54], who associated Basidiomycota with nutrient-rich urban ecosystems.

Importantly, our study identified 83 bacterial and 12 fungal clades as significant class-level biomarkers. Blastochatellia, Holophagae, Thermoanaerobaculia, Vicinamibacteria, Acidobacteriota, and Rubrobacteria were recognized as potential biomarkers, demonstrating significant enrichment across various urban green spaces (Figure 3a). Similarly, Laboulbeniomycetes, Lecanoromycetes, Agaricomycetes, Cystobasidiomycetes, Spizellomycetes, and Mortierellomycota were identified as potential fungal biomarkers significantly enriched in the corresponding green areas (Figure 3b). Our findings are consistent with those of previous studies, indicating that urban green spaces host diverse microbial communities that influence local climatic conditions [10,55,56]. Identifying unique biomarkers for each green space highlights the importance of preserving heterogeneity in urban green space planning to support microbial diversity and ecosystem resilience in urban green spaces.

Microbial diversity is vital for preserving the functions of terrestrial ecosystems [50,57,58]. Interestingly, our results revealed that bacterial communities presented significantly greater alpha diversity in low-intensity human traffic zones in all urban green spaces than in high-intensity human traffic zones, and similar results were observed for fungal communities among the urban green spaces; the alpha diversity was significantly greater in low-intensity zones in forests, wetlands, greenbelts, and parklands, which is in agreement with the findings of previous studies [28,47,59].

The association between phyllosphere microbial diversity and environmental function has recently received substantial attention. The loss of microbial diversity is negatively associated with ecosystem function and can be linked to human health issues [60,61]. In our study, bacterial and fungal diversity were negatively associated with environmental factors, which contrasts with prior studies [62,63]. However, the observed moderate to strong significant associations between bacterial structure and environmental factors such as pH and non-capillary porosity align with previous studies that indicated that pH and nutrient availability are the primary drivers of bacterial community composition [64]. The absence of a significant association between fungal community structure and environmental factors is consistent with prior studies indicating that fungal communities are generally less influenced by soil physicochemical properties [65,66].

Additionally, microbial diversity was greater in low-anthropogenic areas, including forests, wetlands, greenbelts, and parklands. Foot traffic-driven anthropogenic activity indirectly regulates interleaf microorganisms by compacting soil, reducing aeration and water infiltration, and altering soil moisture and nutrient distribution; these changes modify the soil microenvironment, which in turn shifts the composition and activity of microbial communities, including those in the phyllosphere. These patterns may be partly due to differences in soil physicochemical properties, such as soil moisture, which is significantly associated with microbial diversity.

In terrestrial ecosystems, microorganisms play a crucial role in regulating soil nutrient dynamics; however, their aboveground counterparts in urban green spaces remain poorly understood, particularly regarding how vegetation cover and anthropogenic activities shape their communities and associate public risks [56,67]. Our results revealed that the ratios of potential pathogenic bacteria were greater in high-traffic-intensity areas in parklands and greenbelts than in low-traffic-intensity areas; notably, the high relative abundance of Enterococcus in the HIPS zone raises significant public health concerns due to the increasing prevalence of antibiotic resistant strains, especially vancomycin resistant *Enterococcus* (VRE), which complicates treatment and increases the risk of infections, whereas the dominant genera (*Enterobacter* and *Enterococcus*) are significantly associated with the soil moisture content; previous studies have also identified urban runoff as a driver of pathogenic Proteobacteria enrichment in natural watersheds [68,69]. Although the risk of pathogenic bacterial infections in green spaces is relatively low, anthropogenic activity is a critical driver of elevated pathogen abundance, emphasizing the need for targeted management strategies and necessitating methodological advancements to assess health risks comprehensively [70,71].

Co-occurrence network analysis highlighted the distinct interaction dynamics of microbial communities. The bacterial networks presented greater connectivity and modularity, driven by keystone taxa, including the *RCP2-54*, Proteobacteria, Longimicrobia, Blastocatellia, Coriobacteriia, Vicinamibacteria, WCHB1-81, Polyangia, Ignavibacteria, Chloroflexia, Dehalococcoidia, Ktedonobacteri, Myxococcota, Actinobacteria, *WS2* at low-intensity zones for all green space types except wetlands. Conversely, fungal networks presented lower connectivity and modularity, which was centered on Sordariomycetes, Eurotiomycetes, Basidiomycota, Agaricomycetes, Mortierellomycetes, Rozellomycota, Dothideomycetes, Leotiomycetes, Pezizomycetes, Tremellomycetes, Ascomycota, Glomeromycota, Orbiliomycetes, Lobulomycetes, Cystobasidiomycetes taxa. These findings are similar to those of previous studies that documented bacteria as highly interconnected, cooperative communities shaped by keystone taxa [56,72], whereas fungi form modular networks with competitive dynamics that reflect niche differentiation [73].

Therefore, although an in-depth investigation was conducted on the microbial characteristics and potential pathogens in urban green spaces, further research is needed to assess the public health risks posed by these pathogens.

## 5. Conclusions

Anthropogenic activities significantly affect the taxonomic and functional compositions of phyllosphere bacterial and fungal communities. Shifts in the taxonomic composition of bacteria and fungi were due to changes in soil physicochemical properties and human disturbance. In contrast, changes in the functional composition of bacteria were attributed to alterations in soil physicochemical properties and fungal community dynamics, with functional richness being particularly high- in the low-traffic zones of urban green spaces. The species and functional diversity of bacteria and fungi were much greater in low-traffic areas, including forests, wetlands, greenbelts, and parklands, indicating that targeted management practices could increase phyllosphere bacterial and fungal diversity and ecosystem functions. In this study, the risk associated with putative pathogens in urban green spaces was relatively low except for Enterobacter and Enterococcus but cannot be ignored. Anthropogenic activities affected phyllosphere microbial community’s characteristics by altering their physicochemical properties, providing critical insight into urban green spaces management. Future research should focus on identifying specific high-impact activities to refine these management strategies and optimize the balance between human use and the health of urban phyllosphere ecosystems to ensure the sustainability and ecological integrity of urban green spaces.

## Data Availability

The data will be made available upon request from the corresponding authors.

## Supplementary Materials

The following supporting information can be downloaded at https://www.mdpi.com/article/10.3390/microorganisms13112464/s1: Figure S1: Rearification curve for bacteria and fungi across various urban green space types. Figure S2: Representing the relative abundance of bacteria (a), and fungi (b) at initial time at different urban green spaces. Figure S3: Cladogram illustrating the phylogenetic distribution of bacterial (a) and fungal (b) communities across urban green spaces. Figure S4: Correlations between soil physiochemical properties and diversity from floiars. Figure S5: Represent the Network analysis revealing co -occurrence pattern between bacterial classes across different types of urban green spaces. Figure S6: Represent the Network analysis revealing co -occurrence pattern between fungi classes across different types of urban green spaces. Table S1: The basic information of each sampling site; Table S2: Initial foliar microbial alpha diversity for various urban green spaces; Table S3: Bacteria (Sequence processing analysis/sequence denoising or clustering); Table S4: Fungi (Sequence processing analysis/sequence denoising or clustering).
