# Supplementary material for: Foot Traffic Driven Anthropogenic Activity Alters Phyllosphere Microbial Community Characteristics and Putative Pathogens in Subtropical Urban Green Spaces
Source: Microorganisms. 2025 Oct 28;13(11):2464. doi: 10.3390/microorganisms13112464 (PMC12654198; doi:10.3390/microorganisms13112464)
Supplement: Supplementary file 1 [file microorganisms-13-02464-s001.zip › microorganisms-3889744-supplementary.pdf]

Table S1 The basic information of each sampling site.

ID	Longitude (°E)	Latitude (°N)	Elevation (m)	Urban Green Space Types
HIFS1	31.273331	114.236543	144.9	High Intensity Forest
HIFS2	31.272405	114.233944	125.9	High Intensity Forest
HIFS3	31.270369	114.23348	111.3	High Intensity Forest
HIFS4	31.27452	114.239234	242.5	High Intensity Forest
HIFS5	31.272722	114.236976	153.2	High Intensity Forest
HIFS6	31.273621	114.232676	150.5	High Intensity Forest
LIFS1	31.272828	114.242204	288.1	Low Intensity Forest
LIFS2	31.271718	114.24106	245.4	Low Intensity Forest
LIFS3	31.268836	114.237997	163.3	Low Intensity Forest
LIFS4	31.271665	114.242544	287.7	Low Intensity Forest
LIFS5	31.270739	114.242452	264.9	Low Intensity Forest
LIFS6	31.270475	114.244772	272.5	Low Intensity Forest
HIGS1	30.575046	114.377007	28.7	High Intensity Greenbelt
HIGS2	30.561743	114.36705	31.4	High Intensity Greenbelt
HIGS3	30.559526	114.364475	19.1	High Intensity Greenbelt
HIGS4	30.55923	114.362072	19.3	High Intensity Greenbelt
HIGS5	30.557752	114.365162	19.4	High Intensity Greenbelt
HIGS6	30.552431	114.358639	19.7	High Intensity Greenbelt
LIGS1	30.578888	114.392284	18.6	Low Intensity Greenbelt
LIGS2	30.575341	114.392456	18.7	Low Intensity Greenbelt
LIGS3	30.581696	114.393143	18.3	Low Intensity Greenbelt
LIGS4	30.587607	114.395889	18.4	Low Intensity Greenbelt
LIGS5	30.590858	114.398121	18.2	Low Intensity Greenbelt
LIGS6	30.567951	114.397091	18.7	Low Intensity Greenbelt
HIPS1	30.572186	114.339289	18.8	High Intensity Parkland
HIPS2	30.571854	114.340748	18.6	High Intensity Parkland
HIPS3	30.571817	114.342765	18.2	High Intensity Parkland
HIPS4	30.648688	114.331465	26.1	High Intensity Parkland
HIPS5	30.649422	114.333312	25.9	High Intensity Parkland
HIPS6	30.619724	114.318329	21.8	High Intensity Parkland
LIPS1	30.5696	114.342551	18.5	Low Intensity Parkland
LIPS2	30.568417	114.341349	18.3	Low Intensity Parkland
LIPS3	30.5662	114.336028	18.7	Low Intensity Parkland
LIPS4	30.642578	114.334874	21.9	Low Intensity Parkland
LIPS5	30.6394	114.331891	21.2	Low Intensity Parkland
LIPS6	30.642211	114.336863	21.9	Low Intensity Parkland
HIWS1	30.436405	114.282444	26.1	High Intensity Wetland
HIWS2	30.437885	114.28356	22.6	High Intensity Wetland
HIWS3	30.441363	114.285448	18.6	High Intensity Wetland

HIWS4	30.447949	114.285276	16.4	High Intensity Wetland
HIWS5	30.448319	114.289997	21.1	High Intensity Wetland
HIWS6	30.430485	114.281414	24.9	High Intensity Wetland
LIWS1	30.454608	114.284847	20.2	Low Intensity Wetland
LIWS2	30.452684	114.288109	17.3	Low Intensity Wetland
LIWS3	30.451723	114.283045	17.4	Low Intensity Wetland
LIWS4	30.420493	114.278839	21.7	Low Intensity Wetland
LIWS5	30.429375	114.27738	17.8	Low Intensity Wetland
LIWS6	30.419087	114.280985	22.6	Low Intensity Wetland

Table S2. Initial foliar microbial alpha diversity for various urban green spaces (Mean ± SE)

	Bacterial diversity				*Fungal diversity*			
Type of urban green space	***Chao1***	***Observed species***	***Shannon***	***Simpson***	***Chao1***	***Observed species***	***Shannon***	***Simpson***
Forestlands	6713.973±521.662	4317.466±506.898	11.474±0.214	0.989±0.003	296.282±16.707	294.9±16.642	5.456±0.499	0.982±0.005
Greenbelt	2816.63±499.028	2136.466±294.691	10.441±0.103	0.989±0.005	245.325±13.654	225.4±10.567	3.32±0.089	0.43±0.008
Parklands	1695.608±316.828	1178.333±35.114	10.434±0.107	0.988±0.002	163.235±11.075	148.033±6.745	3.248±0.078	0.403±0.089
Wetlands	4625.813±538.41	3282.2±596.811	10.528±0.227	0.988±0.001	264.673±11.483	243.466±11.019	3.671±0.061	0.697±0.063

Table S3. Bacteria (Sequence processing analysis/sequence denoising or clustering). Green space types include forest (F), greenbelts (G), parklands (P) and wetlands (W), with high-intensity (HI) and low-intensity (LI) of human traffic.

Type of UGS	*Input*	*Filtered*	*Denoised*	*Merged*	*Non-chimeric*	*Non-singleton*
**HIFS**	87809	82449	75750	50391	46647	45642
**LIFS**	88271	82612	76110	52994	49908	49149
**HIGS**	96983	90480	83317	59094	55715	54857
**LIGS**	108279	101331	95328	73650	69515	68924
**HIPS**	110049	103119	96954	73775	69254	68724
**LIPS**	110352	101701	99578	92872	85781	85614
**HIWS**	109458	101313	98549	90364	83250	83001
**LIWS**	108523	100255	96876	81868	68508	67868
**Total**	**819727**	**763262**	**722464**	**575010**	**528579**	**523780**

Table S4. Fungi (Sequence processing analysis/sequence denoising or clustering). Green space types include forest (F), greenbelts (G), parklands (P) and wetlands (W), with high-intensity (HI) and low-intensity (LI) of human traffic.

Type of UGS	*Input*	*Filtered*	*Denoised*	*Merged*	*Non-chimeric*	*Non-singleton*
**HIFS**	82266	70174	69605	66684	64166	64166
**LIFS**	82545	73033	72361	70422	66375	66374
**HIGS**	102676	94588	93942	91750	86379	86379
**LIGS**	125993	112377	111761	103590	94962	94962
**HIPS**	120186	109404	108671	101874	93304	93304
**LIPS**	108911	101126	100732	96945	88210	88210
**HIWS**	124277	112502	111806	109139	98020	98020
**LIWS**	129401	119462	118947	117383	107617	107617
**Total**	**876257**	**792667**	**787827**	**757790**	**699035**	**699035**

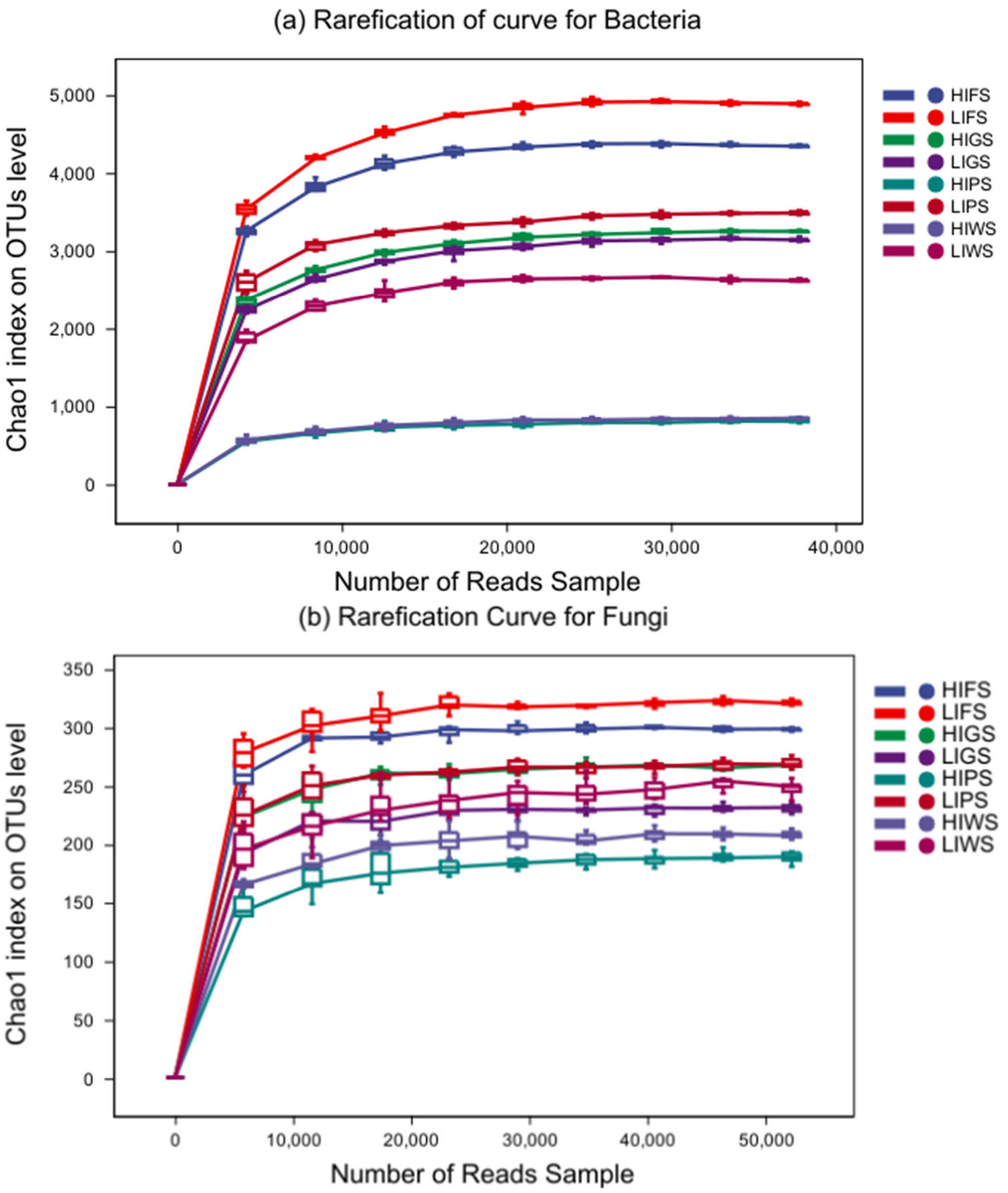

Fig.S1. Rearification curve for bacteria and fungi across various urban green space types. Green space types include forests (FS), greenbelts (GS), parklands (PS), and wetlands (WS), with high-intensity (HI) and low-intensity (LI).

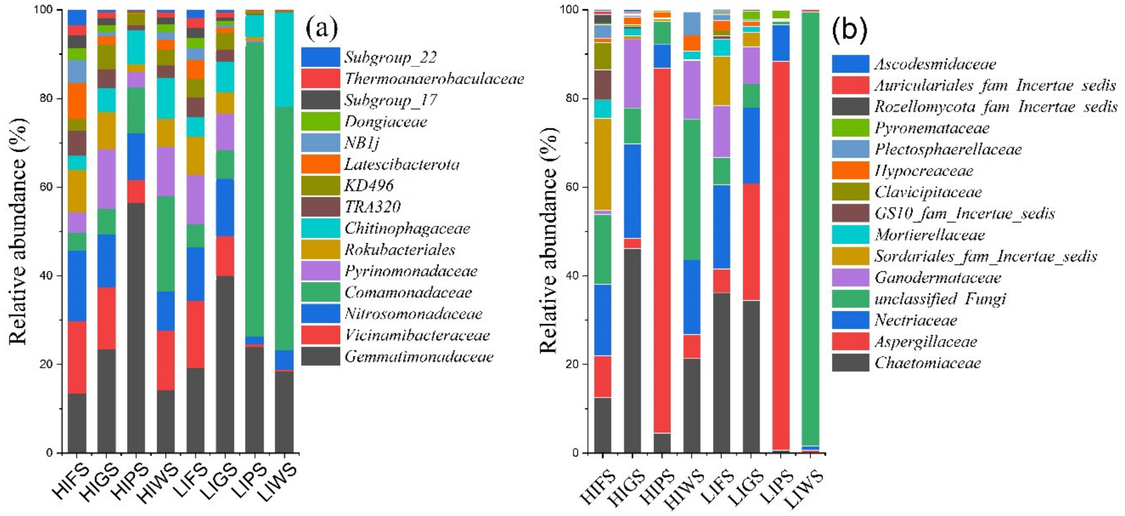

Fig.S2. Representing the relative abundance of bacteria (a), and fungi (b) at initial time at different urban green spaces. Green space types include forests (FS), greenbelts (GS), parklands (PS), and wetlands (WS), with high-intensity (HI) and low-intensity (LI).

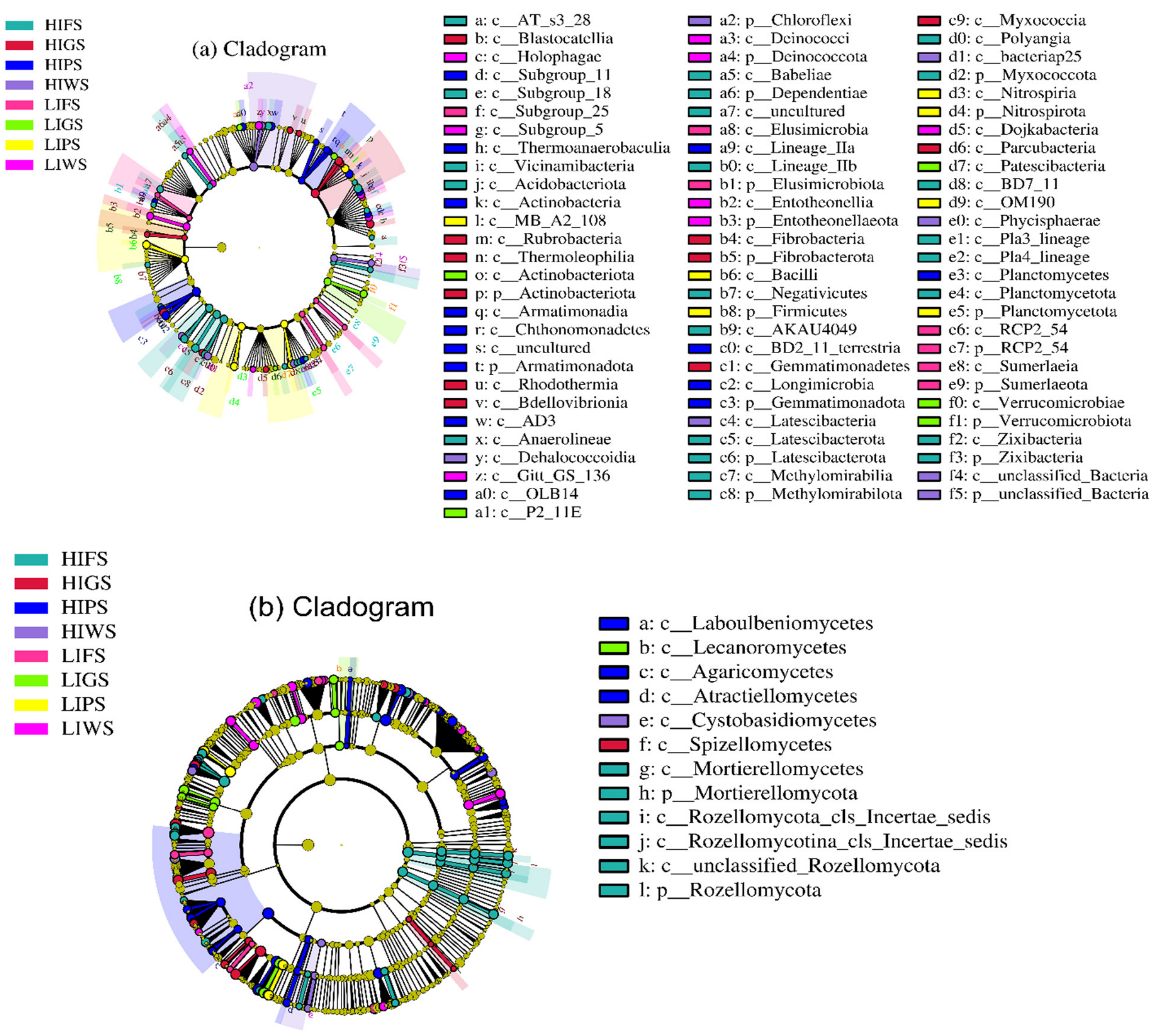

Figure.S3. Cladogram with linear discriminant analysis (LDA) scores >3.0, illustrating the phylogenetic distribution of bacterial (a) and fungal (b) communities across urban green spaces. The diameter of each circle corresponds to the relative abundance of the taxon. Circles represent phylogenetic levels from phylum (innermost) to class (outermost). p: phylum; c: class. Green space types include forests (FS), greenbelts (GS), parklands (PS), and wetlands (WS), with high-intensity (HI) and low-intensity (LI).

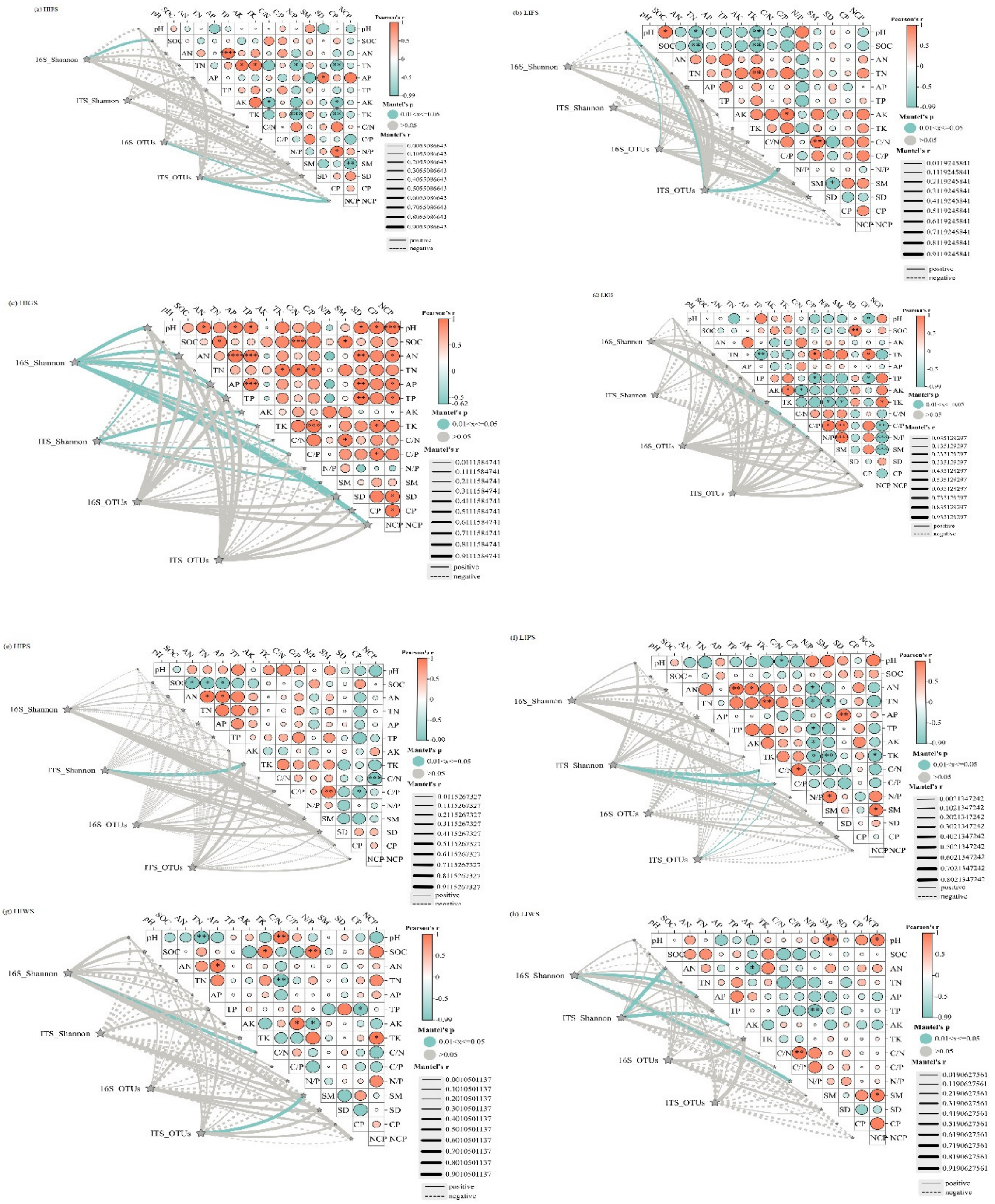

Figure.S4. Correlations between soil physiochemical properties and diversity from Leaves. Colors indicate correlation types. Line width corresponds to the partial Mantel's r statistic. Pairwise comparisons of environmental factors are also shown, with a color gradient denoting Pearson's correlation coefficient. Asterisks indicate the statistical significance (***p < 0.001; **p < 0.01; and * p < 0.05). Green space types include forests (FS), greenbelts (GS), parklands (PS), and wetlands (WS), with high-intensity (HI) and low-intensity (LI).

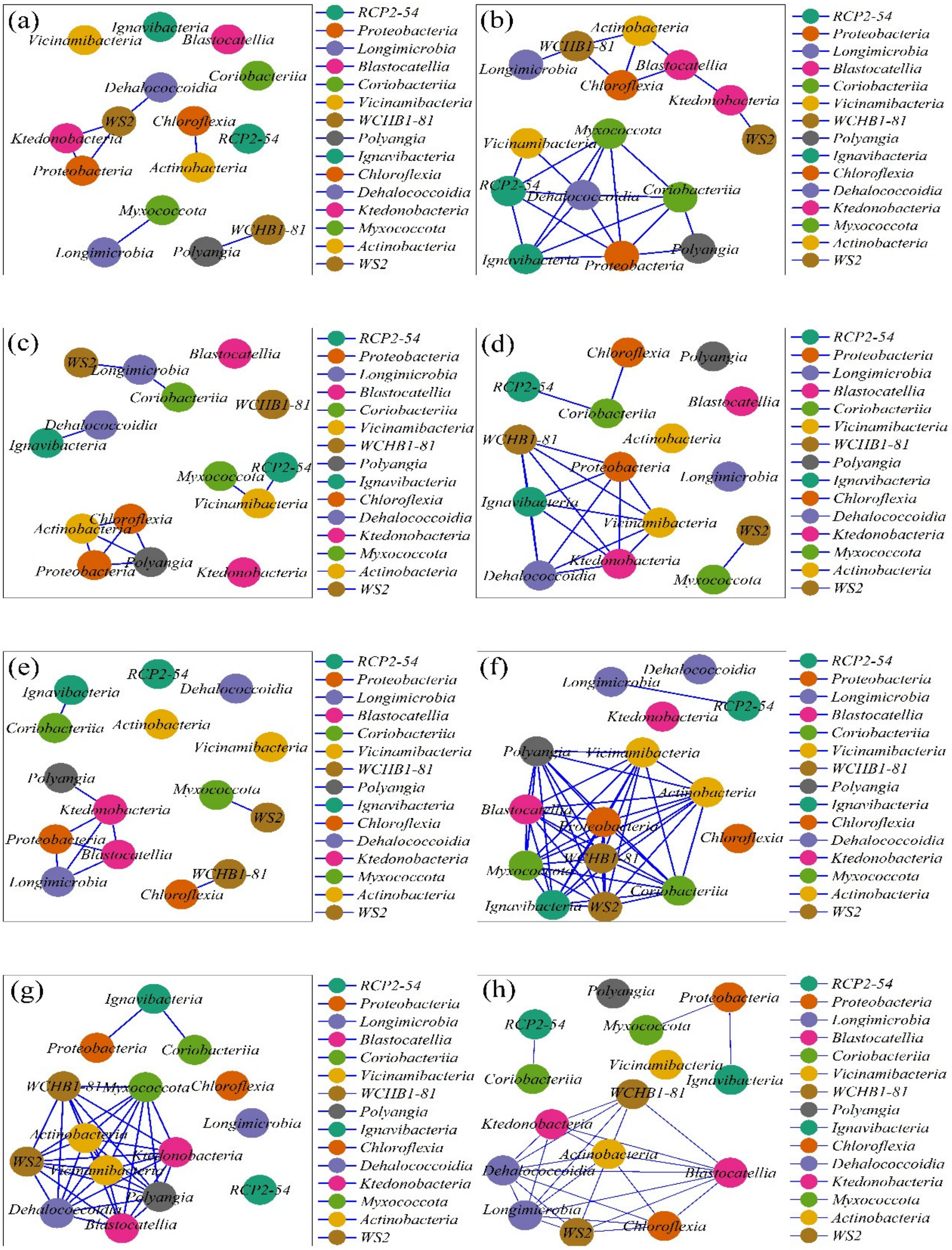

Figure.S5. Represent the Network analysis revealing co -occurrence pattern between bacterial classes across different types of urban green spaces. Each node represents a bacterial class, and nodes are color coded according to the top 15 class. While edges are dependent on the correlation coefficients. (a) forest high intensity, (b) forest low intensity, (c) greenbelt high intensity, (d) greenbelt low intensity, (e) parkland high intensity, (f) parkland low intensity, (g) wetland high intensity, and (h) wetland low intensity. Green

space types include forests (FS), greenbelts (GS), parklands (PS), and wetlands (WS), with high-intensity (HI) and low-intensity (LI).

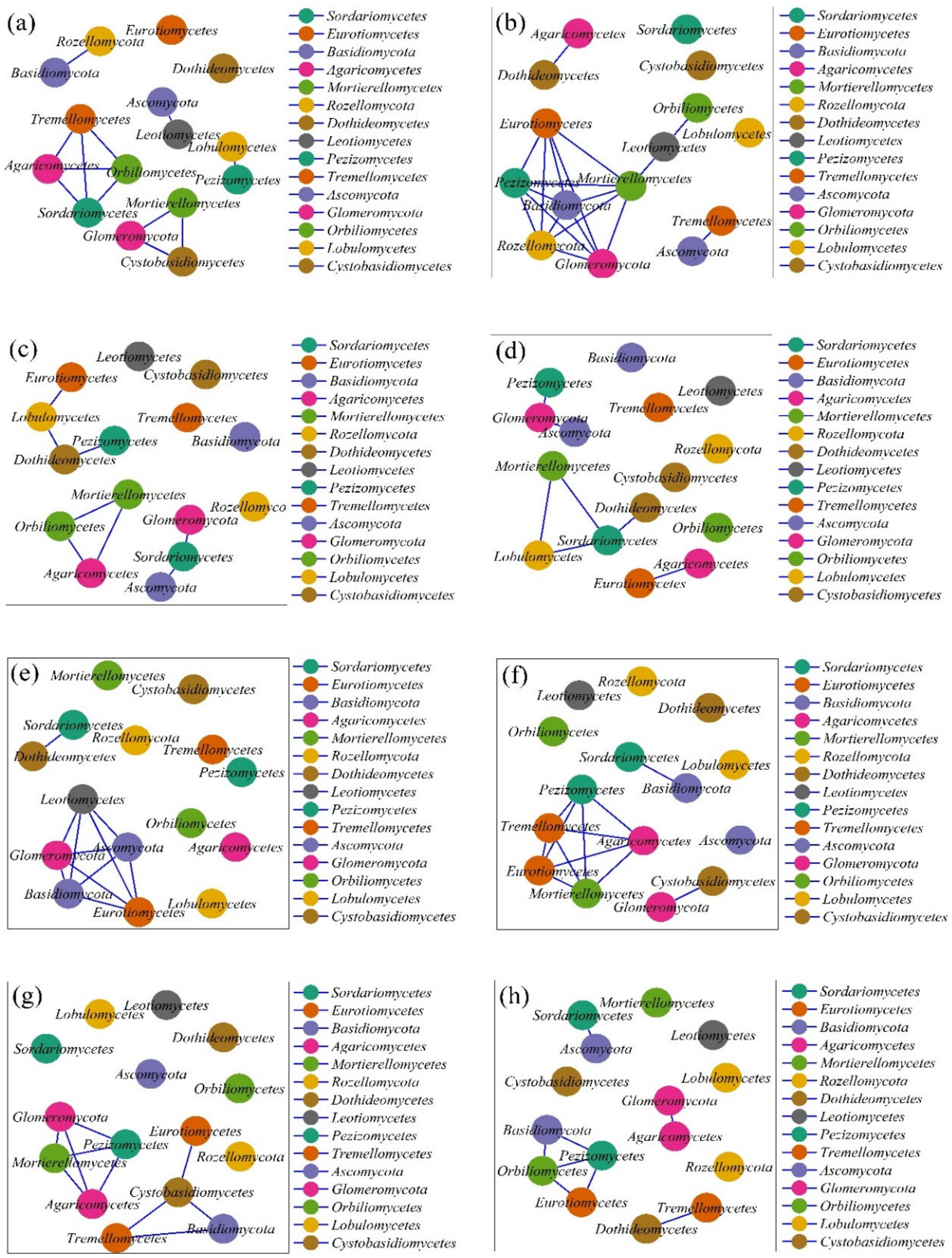

Figure. S6. Represent the Network analysis revealing co -occurrence pattern between fungi classes across different types of urban green spaces. Each node represents a bacterial class, and nodes are color coded according to the top 15 class. While edges are dependent on the correlation coefficients. (a) forest high

intensity, (b) forest low intensity, (c) greenbelt high intensity, (d) greenbelt low intensity, (e) parkland high intensity, (f) parkland low intensity, (g) wetland high intensity, and (h) wetland low intensity. Green space types include forests (FS), greenbelts (GS), parklands (PS), and wetlands (WS), with high-intensity (HI) and low-intensity (LI).
